# Reply to ‘Comment on ‘BAG-1 as a biomarker in early breast cancer prognosis: a systematic review with meta-analyses’’

**DOI:** 10.1038/s41416-018-0032-y

**Published:** 2018-03-15

**Authors:** E. S. Papadakis, T. Reeves, N. H. Robson, T. Maishman, G. Packham, R. I. Cutress

**Affiliations:** 1 Cancer Research UK Centre Cancer Sciences Unit, Southampton General Hospital, Tremona Road, Southampton, SO16 6YD UK; 2 Southampton Clinical Trials Unit, University of Southampton, Southampton, SO17 1BJ UK; 3 University Hospital Southampton, University of Southampton Faculty of Medicine, Southampton General Hospital, Tremona Road, Southampton, SO16 6YD UK

**Keywords:** Biocatalysis, Carbohydrates

We thank Sauerbrei and Haeussler^1^ for their interest in our paper^2^ and their methodological comments. Our aim was to undertake these analyses and draw some conclusions from the available literature regarding the prognostic significance of BAG-1 in breast cancer, given multiple studies. We felt that this was important, particularly since BAG-1 is already included in multi-gene assays widely used as part of routine clinical practice and due to ongoing investigation into the possibility of inhibition of BAG-1 function as a potential therapeutic strategy. We did not use the supplementary information provided within the REMARK Explanation and Elaboration paper.^3^ We provided our interpretation whether details within the REMARK checklist were included in the papers and, as we highlighted, the literature reviewed was heterogeneous and with a range in study quality. We believe our conclusions were drawn with appropriate caveats to highlight this.
